# Diverse arachnoid cyst morphology indicates different pathophysiological origins

**DOI:** 10.1186/2045-8118-11-5

**Published:** 2014-03-03

**Authors:** Katrin Rabiei, Magnus Tisell, Carsten Wikkelsø, Bengt R Johansson

**Affiliations:** 1 Institute of Neuroscience and Physiology, c/o Neurosurgical Clinic, Sahlgrenska University Hospital, Gothenburg SE-413 45, Sweden; 2 Neurosurgical Clinic, Sahlgrenska University Hospital, Gothenburg SE-413 45, Sweden; 3 Institute of Neuroscience and Physiology, Sahlgrenska Academy, University of Gothenburg, P.O. Box 430, Gothenburg SE-40530, Sweden; 4 The Electron Microscopy Unit, Institute of Biomedicine, Sahlgrenska Academy, University of Gothenburg, P.O. Box 440, Gothenburg SE-40530, Sweden

**Keywords:** Arachnoid cyst, Subarachnoid cyst, Fenestration surgery, Electron microscopy, Meningothelium, Ependyma, Chiari arachnoid

## Abstract

**Background:**

There are few, limited, and to some extent contradictory, reports on the cellular and subcellular morphology of arachnoid cysts. In the literature cyst membranes are described as similar to, or as vastly different from, normal arachnoid membranes.

**Methods:**

This paper reports electron microscopic analyses of symptomatic cysts from 24 patients (12 males and 12 females; age 10–79), that underwent fenestration surgery. Fourteen cysts were located in the middle cranial fossa (temporal), one in the interpeduncular cistern, five in the posterior fossa, and four were overlying the frontal cortex.

**Results:**

Microscopic findings confirmed the diverse nature of this clinical condition. Twelve cyst walls resembled normal arachnoid, four had a conspicuous core of dense fibrous tissue with a simple epithelial lining, and the remaining aberrant cysts exhibited non-arachnoid luminal epithelia with plentiful microvilli and/or cilia, and also nervous tissue components in the wall. The possible identity and origin of various cyst types are discussed. We hypothesize that cysts are formed mostly at an early stage of embryonic development, as a teratological event.

**Conclusions:**

Cysts with various epithelial linings and extracellular components most likely have different barrier properties and fluid turnover characteristics. Further studies are needed to elucidate relations between cyst morphology, fluid composition, pathogenesis, and clinical behaviour including growth rate and relapse tendency.

## Background

Arachnoid cysts (AC) are fluid filled cyst-like structures that are anatomically connected with the arachnoid mater and can be found throughout the cranial-spinal axis with a preponderance in the middle cranial fossa [1]. Several mechanisms have been proposed to explain their formation such as splitting or duplication of the arachnoid membrane [2-4], trauma [5,6], and in a few cases genetic factors [7-11]. Some of these cysts expand over time which has variously been explained by fluid entering through one-way valves [12-15], due to fluid secretions by cells lining the cyst lumen [16,17], or by osmosis [18].

The ultrastructure of the arachnoid cyst membrane has been sparsely studied previously. Rengachary and Watanabe described the ultrastructure of three arachnoid cysts and found the cyst wall to be very similar to normal arachnoid mater [2]. A cyst membrane similar to normal arachnoid tissue was also reported by Go *et al*. [16,19]. However, there have also been reports of cyst membranes containing other types of tissue, e.g. choroid plexus and respiratory-like epithelium [20-23]. We decided to study the morphology of symptomatic arachnoid cysts with the main focus on electron microscopy in 24 consecutive cases operated in our department.

## Methods

The study was performed as a prospective explorative study. It was approved by the Regional Ethical Committee (380–09) and all patients gave their written consent to participate in the study. In the pediatric cases written consent was given by the patients’ parents.

Twenty-four consecutive patients (12 males and 12 females) with symptomatic ACs, who after evaluation were offered and accepted surgical treatment for their cysts, were included. Patients were both pediatric and adult cases, ages ranging from 10 to 79 years. Fourteen cysts were located in the middle cranial fossa (temporal, 11 in the left and 3 in the right), one in the interpeduncular cistern, five in the posterior fossa, and four were overlying the frontal cortex (see Table 1).

**Table 1:** Demographic and clinical data in relation to cyst morphology

**Group**	**Arachnoid cysts**	**Connective tissue cysts**	**Aberrant cysts**
No. of cases (with head trauma)	12 (0)	4 (1)	8 (3)
Age; range and median (years)	10-67 (34)	34-73 (50)	15-79 (30)
Sex	7 F	1 F	4 F
	5 M	3 M	4 M
Location	8 Temporal	4 Temporal	2 Temporal
	2 Frontal		2 Frontal
	2 Posterior fossa		3 Posterior fossa
			1 Interpeduncular cistern

**Figure 1:**
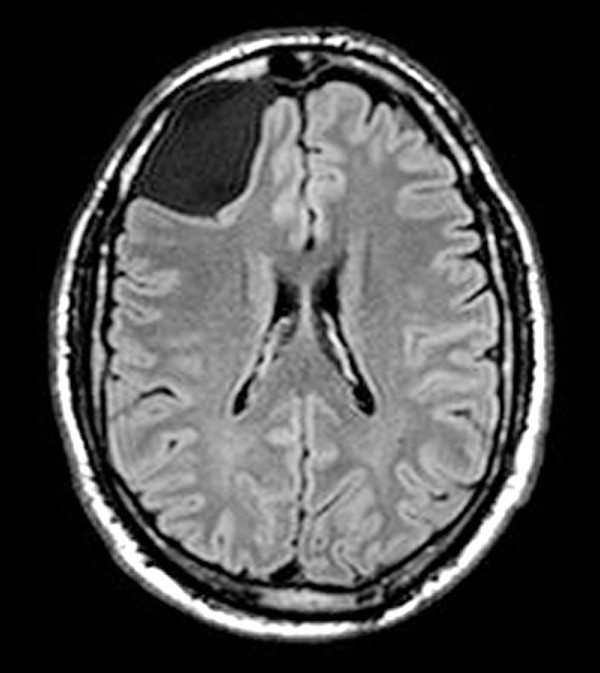
**T1-weighted MRI image.** A large arachnoid cyst deforms right frontal lobe; outer membrane of cyst is faintly visible. This patient presented with headache, vertigo, and seizures. The cyst wall consisted of respiratory-like epithelium.

All subjects had a thorough examination in our clinic for hydrocephalus and cyst patients. Patients were evaluated by a neurologist and a neurosurgeon and a full history according to a special cyst protocol was taken by the neurologist. Cyst protocol included history of complications during birth as well as medical issues, surgical history, medications, any head trauma during life and admissions to hospital. If head trauma was reported, patients were asked about loss of consciousness and/ or memory loss along with any symptoms after the head trauma. All patients were also subject to physiotherapeutic and neuropsychological testing, as well as MRI, radionuclide cisternography (RC), and lumbar puncture sampling of 10 mL CSF before surgery. The MRI protocol included flair-, diffusion- and T1-weighted sequences for closer differential diagnosis of the cysts. The T1 scan was to determine cyst volume (Figure 1). The patients presented with various symptoms assumed to relate to the presence of the cyst, e.g. headache, dizziness, seizures, focal neurological symptoms, and signs of cognitive impairment. Demographic data are presented in Table 1.

Patients were operated under general anaesthesia with a craniotomy and microsurgical fenestration of the cyst wall. The procedure commenced with puncture and cyst fluid collection followed by excision of part of the cyst wall. This membrane was subjected to microscopical analyses. All patients were re-examined 3 months after surgery with the same test battery and MRI. Reference (control) material was obtained from normal-looking arachnoid mater from the cisterna magna of 5 consecutive patients with Chiari type I malformation undergoing first surgery (3 females, 2 males, age 19–61 years). The surgical samples were divided into two parts. One small piece, for routine histopathological diagnosis, was fixed in 4% formaldehyde; no material was reserved for immunohistological analyses. The major part was immersed for 24 to 72 h in a mixture of 2% formaldehyde + 2.5% glutaraldehyde + 0.02% sodium azide in 0.05 M Na cacodylate buffer, pH 7.2. This sample was prepared for transmission and scanning electron microscopy (TEM and SEM). Pieces intended for TEM were postfixed in 1% osmium tetroxide + 1% potassium hexacyanoferrate in 0.1 M cacodylate followed by *en bloc* staining with uranyl acetate and dehydration in ethanol. Specimens were infiltrated with epoxy resin and cured by heat according to routine methods. Sections were cut with a Leica UC6 ultramicrotome (Leica Microsystems, Vienna, Austria) fitted with diamond knives. Semithin sections 1 μm thick were examined by light microscopy after treatment with Richardson’s stain (0.5% Azur II and 0.5% Methylene blue). Ultrathin sections were contrasted with uranyl acetate and lead citrate before examination in a digitized LEO 912AB Omega electron microscope (Carl Zeiss SMT, Oberkochen, Germany). Digital image files were acquired with a MegaView III or Veleta CCD camera (Olympus-SiS, Münster, Germany). For samples prepared for SEM the aldehyde fixation was followed by repeated osmification according to the OTOTO protocol [24]. Specimens were then dehydrated in ethanol, ending in hexamethyldisilazane, which was allowed to evaporate. They were mounted on aluminum stubs and sputter coated with palladium before examination in a Zeiss 982 Gemini field emission scanning electron microscope (Carl Zeiss).

## Results

### Clinical outcome

Patient history revealed no instances of complications during birth or early childhood. Only four patients reported head trauma. All head traumas were minor and there was no correlation between head trauma and cyst location or type (Table 1). All patients improved after cyst fenestration as determined at follow-up. Three patients had temporary postoperative complications; one had meningitis, one status epilepticus and one a transient postoperative aphasia

### Neuropathological examination

Samples from 20 patients underwent routine pathological-anatomical diagnosis (four were omitted for technical reasons). The structure of these samples was considered to agree with arachnoid cyst morphology.

### Detailed morphology – general cyst wall composition

**Figure 2:**
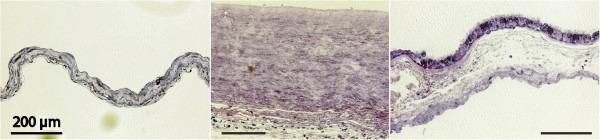
**Light microscopic overviews of 1 μm sections of cyst walls.** Left to right: arachnoid, fibrous, and aberrant types, the latter with ciliated epithelium. Cyst lumen top, dural side below, Richardson’s stain, all scale bars = 200 μm.

A macroscopically-evident variation in cyst wall character was confirmed by subsequent light microscopy of 1 μm sections of plastic-embedded specimens. Cyst wall thickness ranged from <10 μm to ≈ 800 μm and there were considerable differences in cellularity, connective tissue elements and vascularisation (Figure 2). This diversity was further revealed by the increased resolution of scanning and transmission EM. Based on the EM findings of cyst wall tissue, we tentatively grouped the samples into three main categories (Tables 1 and 2). This division was arbitrary and depended on recognizable and dominant features and it should be noted that individual cysts displayed regions that did not comply with this provisional classification. There was no correlation between the location of the cysts and their morphology.

i) Cysts composed of arachnoid-like tissue (12 cases).

**Table 2:** Summary of the morphological characteristics of cysts in the respective groups

**Group**	**Arachnoid cysts**	**Connective tissue cysts**	**Aberrant cysts**
Thickness variation	20-150 μm	30-800 μm	6-400 μm
Connective tissue layer	Thin	Thick; major part of cyst wall	Thin
Meningothelial epithelium	Dominant on both sides	Sparsely present	Sometimes present on dural side
Luminal epithelium	One to several layers	Single layer	Stratified or single layer
Luminal cilia	Not present	Not present	Often present
Luminal microvilli	Sparse	Sparse	Abundant
Junctional complexes	Tight and adherence junctions, desmosomes	Simple	Elaborate; tight, adherence, and gap junctions, desmosomes
Glial cell processes	Not present	Not present	Often present
Neuropil	Not present	Not present	Present in one cyst

**Figure 3:**
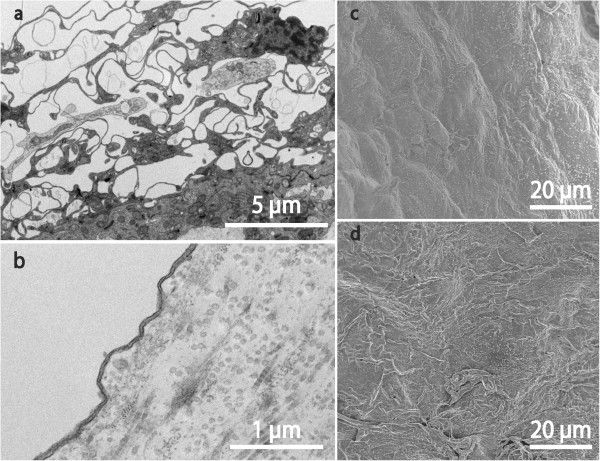
**EM findings on arachnoid and fibrous cysts.** TEM of dural aspects of arachnoid cyst **(a)** and fibrous cyst **(b)**; SEM of luminal surface of arachnoid cyst **(c)** and of fibrous cyst **(d)**. A typical arachnoid organisation is seen in **(a)** with highly complex cellular extensions in whorls encircling wide intercellular spaces. In **(b)** only a single extremely attenuated cell covers a matrix rich in collagen and ground substance. In **(c)** and **(d)** scattered microvilli are seen as bright spots. The luminal surface epithelium is smooth in **(c)**, more irregular with some overlapping or desquamating cells in **(d)**.

A multilayered subdural meningothelium formed very complex cellular extensions in whorls and wide extracellular spaces (Figure 3a). In spite of these elaborate cell interactions the *en face* SEM examination as a rule revealed a smooth and structure-less continuous surface (not shown). The core of the cyst wall consisted of a trabecular connective tissue with widely spaced cells and scattered microvessels. The cyst lumen (Figure 3c; SEM image) was mostly lined by a single layer of flattened epithelial cells with organized junctions and a moderate number of short microvilli. Regions of multilayered arachnoid epithelium also occurred.

ii) Cysts composed of fibrous connective tissue (4 cases).

These cyst walls were generally thicker than in the other groups and had a dominating core of dense connective tissue with scattered cellular elements. The epithelium on both aspects of the cyst was single-layered (Figure 3b) except for a few strands of meningothelial appearance on the subdural side. The luminal aspect could indicate some desquamation of cells and showed few microvilli (Figure 3d).

iii) Cysts with aberrant structure (8 cases).

**Figure 4:**
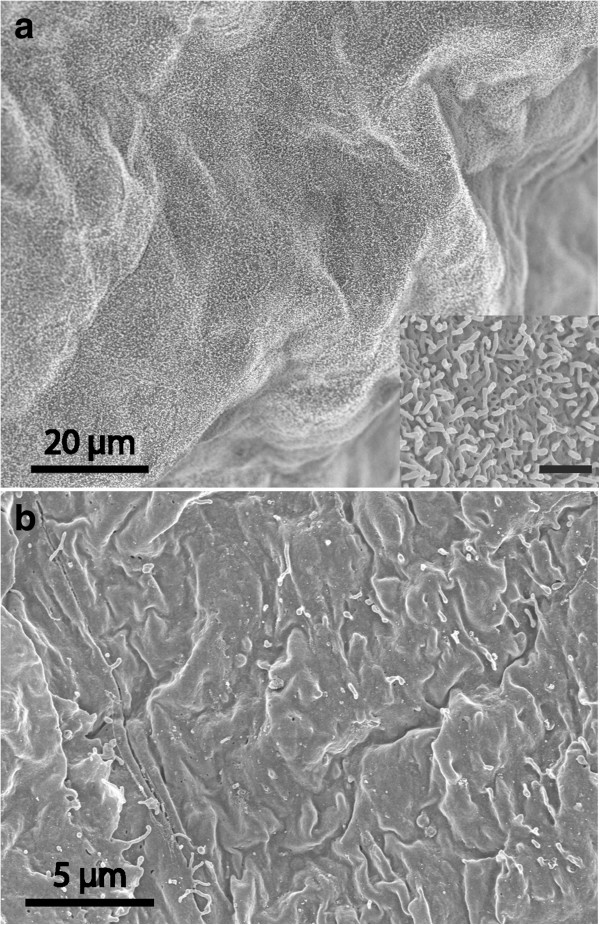
**Aberrant-type cyst.** SEM micrographs of cyst with uniform appearance at both luminal **(a)** and dural surfaces **(b).** Luminal surface with dense distribution of short microvilli (see insert; bar 1 μm). The dural-aspect cells appear to be arranged in a tile-like manner and carry few microvilli.

**Figure 5:**
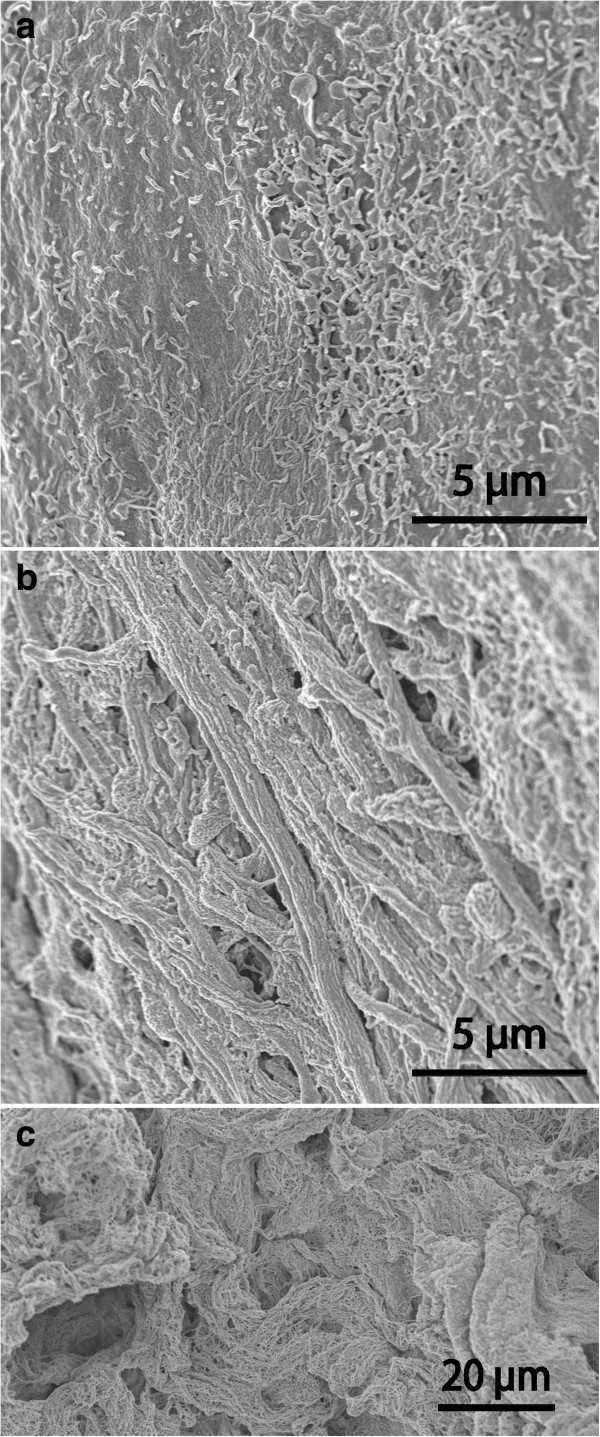
**Aberrant-type cysts.** A comparison between luminal **(a)** and dural **(b)** cyst wall in a case where the SEM architecture of the dural side was particularly complex. Luminal cells **(a)** form a smooth surface with intermediate density of microvilli. Note elongated strands of cellular irregularities giving an uneven webbed dural surface **(b)**. In comparison, **(c)** depicts an isolated sample where connective tissue filament bundles were exposed in the absence of covering cells on the luminal surface.

**Figure 6:**
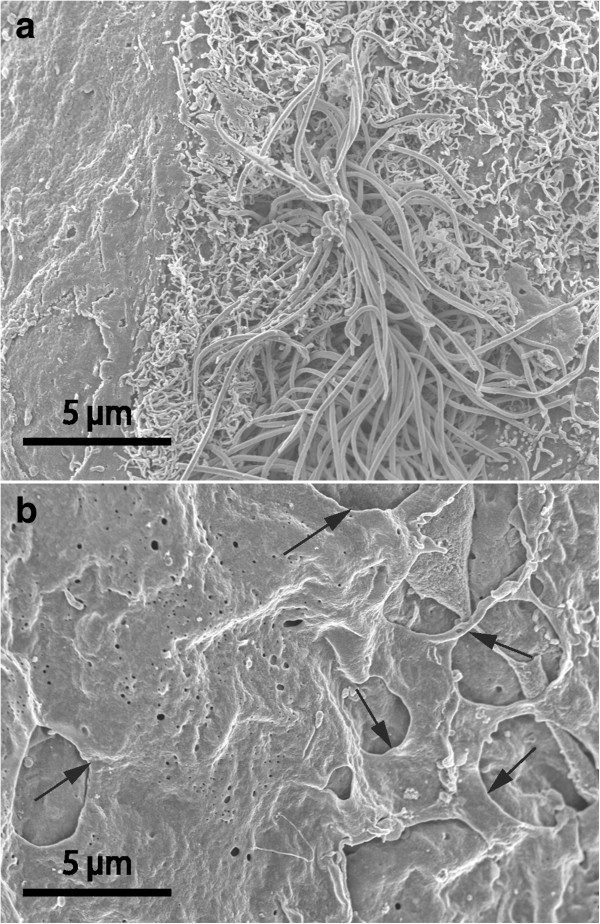
**Aberrant-type cyst, SEM images.** On luminal aspect **(a)** isolated ciliated cells were observed, here a microvillus-rich cells with a central tuft of cilia is surrounded by a profile-poor flat luminal epithelium. The dural surface **(b)** displayed numerous fine-edged openings (arrows) formed by cellular extensions and connections.

**Figure 7:**
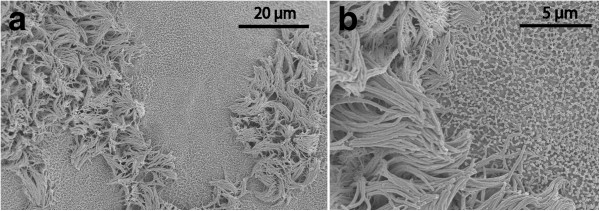
**Aberrant-type cyst, SEM recordings of luminal surface.** The epithelium exhibits a mosaic of two cell types, one equipped with numerous cilia, the other with extensive microvilli **(a)**. On detailed examination **(b)**, the bundles of long cilia are mixed with slender cellular projections (upper and lower left corners).

**Figure 8:**
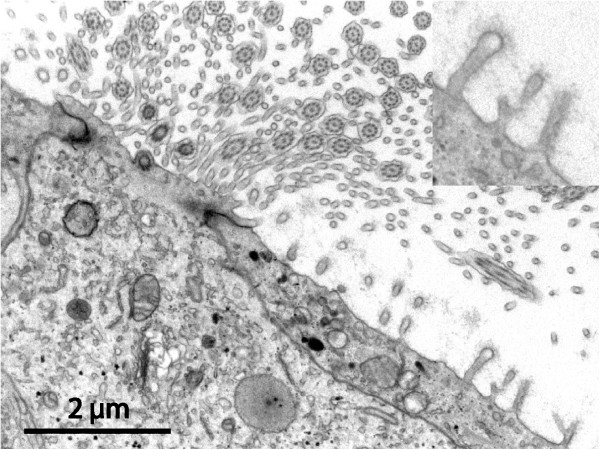
**Aberrant-type cyst luminal surface.** TEM micrograph of apical region of a high ciliated epithelial cell surrounded by flat microvilli-carrying cells (same specimen as in Figure 7). The two cell types meet in well-developed junctions. The blunt microvilli in the epithelial cell to the right have a constant length ending in a bulb-like widening, and carry a conspicuous fuzzy coat covering their surface (insert). Note also that the ciliated cell in addition to the obvious cilia also possesses numerous long and slender cytoplasmic extensions (mostly cross-sectioned) with a smaller diameter than the microvilli of the neighbouring cell.

**Figure 9:**
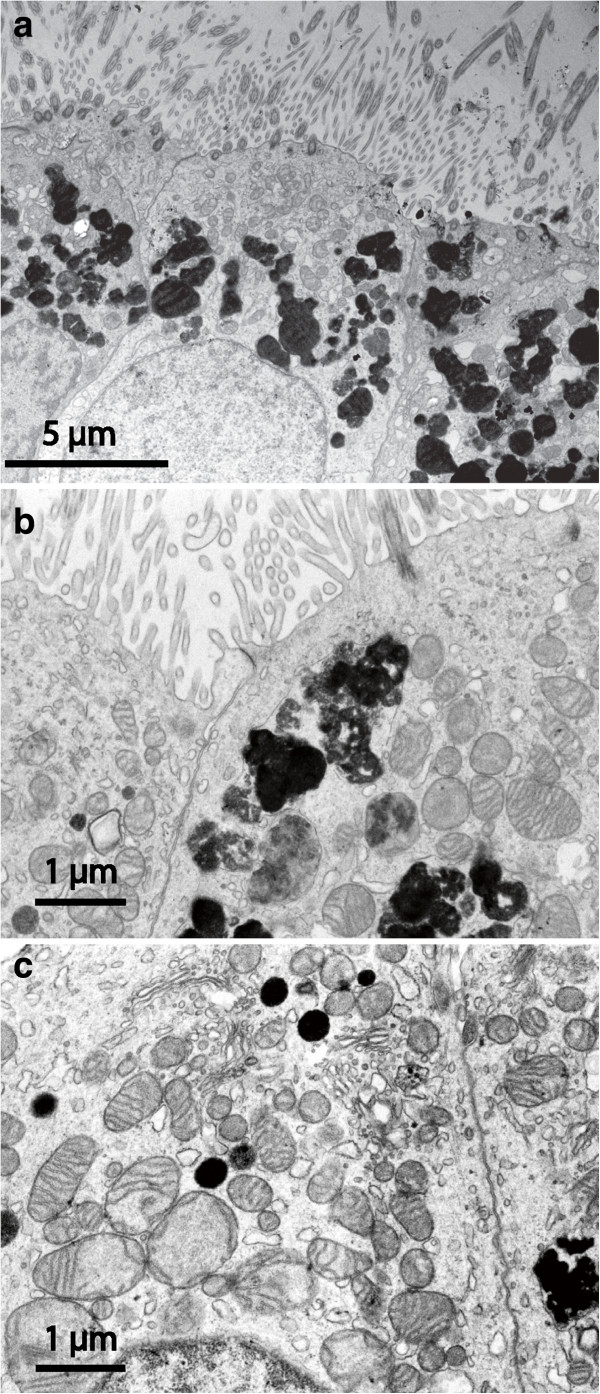
**Aberrant-type cyst: TEM details of luminal ciliated epithelium.** In **(a)** and **(b)** slender long apical microvilli are more numerous than cilia. In the cytoplasm numerous irregular vesicles are seen with dense osmophilic content at various degrees of packing. The same cytoplasmic region is rich in mitochondria. Epithelial cells are closely adherent with probable tight junction complexes next to cyst lumen. In **(c)** a number of spherical vesicles with electron dense homogenous content are interspersed with numerous mitochondria.

These samples deserve a more detailed description since they presented individually unique features. Most cysts in this heterogeneous category had a luminal surface that was richly equipped with microvilli of uniform length (Figure 4). One of these cases showed a unique SEM architecture on the dural aspect (Figure 5). In four cysts the luminal epithelium also contained ciliated cells. In one sample the ciliated cells were few and isolated in an environment of microvillus-rich cells (Figure 6). In three other cysts, ciliated cells occupied a more prominent fraction of the luminal surface and were distributed as a mosaic of islands mixed with microvillus-rich cells (Figure 7), or formed an almost complete luminal covering. From their apical surface ciliated cells also projected long slender microvilli which lacked the surface coating of the uniform blunt microvilli in neighbouring cells (Figure 8). The apical cytoplasm of ciliated cells was rich in mitochondria. In one cyst the ciliated luminal epithelium in addition exhibited apical accumulations of possible secretory granules with a very electron-dense content (Figure 9). The true nature of this material could not be determined further by EM. As a rule, ciliated cells were incorporated into an epithelium with several cell layers and well-developed intercellular junctional complexes were evident (Figures 8 and 9).

**Figure 10:**
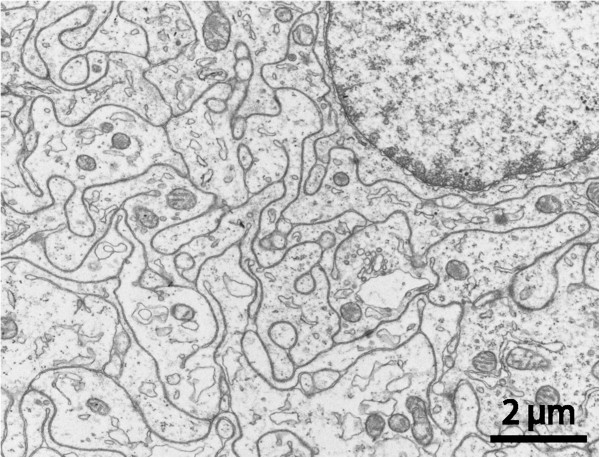
**Aberrant-type cyst. **TEM section from cyst wall with numerous tightly interlocking cell processes containing microtubules, filaments, scattered vesicles and some mitochondria, i.e. exhibiting neuropil morphology. Occasional myelinated fibers were encountered (not shown).

**Figure 11:**
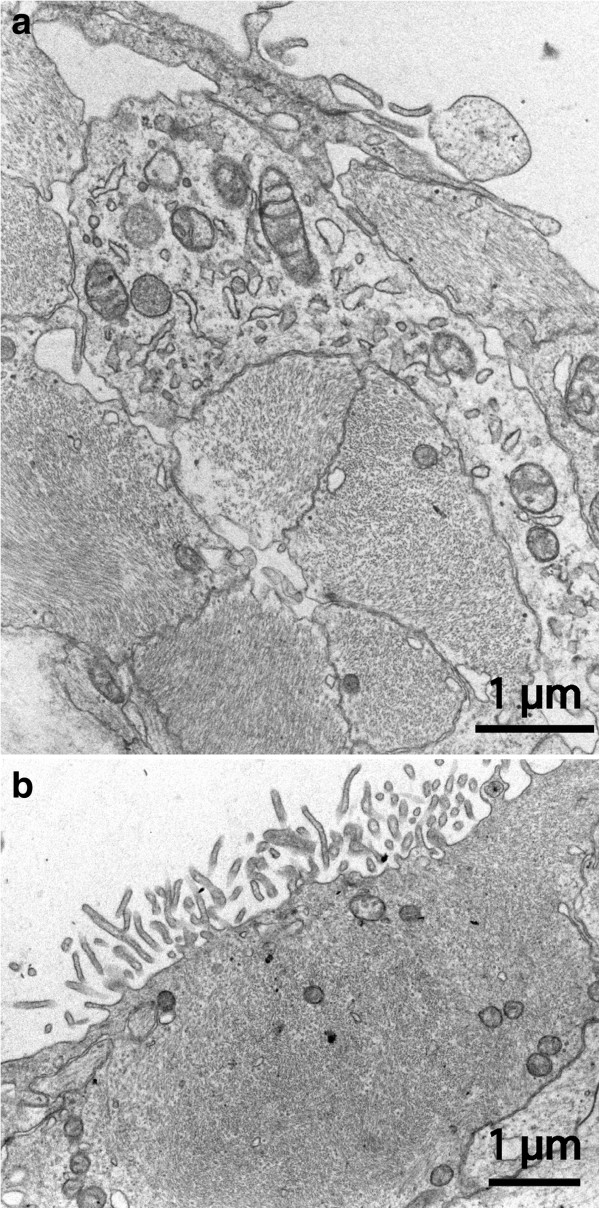
**Aberrant-type cyst.** TEM micrographs from the luminal region of a cyst showing tightly packed cell profiles where the cytoplasm was almost completely occupied with masses of intermediate filaments. In **(a)** an organelle-rich cell is squeezed between filament-rich cells in both epithelial and sub-epithelial position, **(b)** shows a filament-rich cell with brush border-like microvilli towards cyst lumen.

One cyst located in posterior fossa, had sub-epithelial tissue of neuropil appearance, i.e. a complex packing of unmyelinated nerve fibres (Figure 10). This tissue component had a patchy distribution. In three cases elaborate networks of cellular processes with highly-condensed masses of cytoskeletal fibrils indicating a glial nature, formed a substantial part of the cyst wall. These cells were mostly sub-epithelial but could also extend into a lumen-lining position (Figure 11).

### Detailed morphology – barrier-mediating structural features of cysts

**Figure 12:**
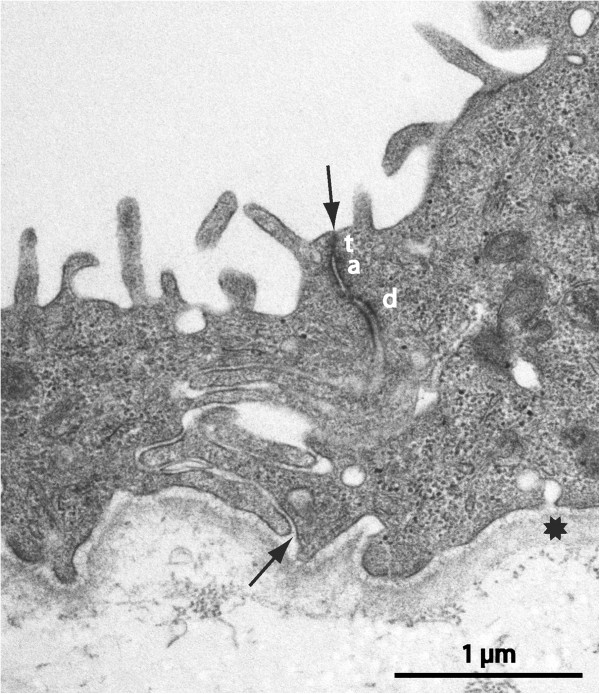
**Fibrous-type cyst, TEM micrograph.** Junctional region of monolayered luminal epithelium, same specimen as Figure 3d. A complex, meandering, intercellular cleft runs between arrows; lumen up. Tight (t) and adherens (a) junction zones and a desmosome (d) are indicated. There is a distinct, continuous basal lamina (star).

The luminal cyst epithelium constitutes a primary barrier for cyst fluid exchange. Regardless of type (low, high, single or multi-layered, ciliated &c.) the epithelial lining was ordered and continuous in the examined surfaces of all samples except one. The latter refers to one specimen where a denuded area exposing the underlying collagenous core was encountered in the SEM (Figure 5c). However, this might represent an artefact from harvest or preparation. Intercellular junctions showed the expected variations from the simple abutting in very low squamous epithelia to the elaborate multiple junctional elements in high and multilayered epithelia (Figures 8 and 9). In favourable sections the various subdivisions of junctional elements could be identified (Figure 12). A sub-epithelial basal lamina was always present (Figure 12) but occasionally was not perfectly continuous. We did not observe indications of inflammatory activity in any specimen.

### Arachnoid membrane from Chiari patients

**Figure 13:**
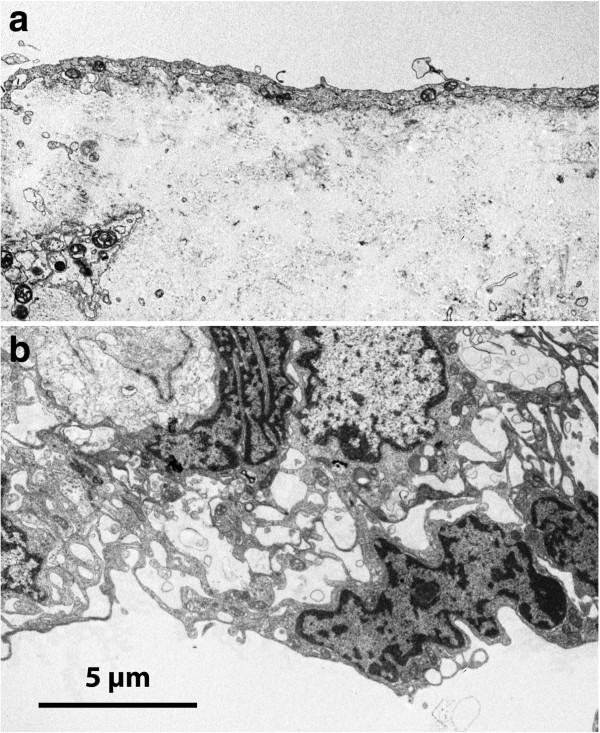
**TEM of comparison arachnoid from a patient with Chiari type I malformation.** On pial aspect **(a)** an attenuated single-layered epithelium covers a cell poor matrix; the cellular arrangement towards the dura **(b)** is in agreement with normal arachnoid with the superficial cells forming complicated extensions in a network manner (cf. Figure 3a).

Samples from this patient group do not constitute an ideal control for normal arachnoid tissue. Nevertheless, these membranes agreed well with the expected morphology with a thickness up to 60–65 μm and organisation into three layers [2]. Meningothelial epithelium was observed on the dural and pial sides with an organisation similar to that of the cysts described as arachnoid-like above (Figure 13). Generally, the intervening connective tissue stroma presented a more-developed three-dimensional network of connective tissue cells than did the cyst samples of the arachnoid tissue category. In the SEM (not shown) the pial epithelium had scattered stubby microvilli while the dural side was mostly smooth with no cellular projections.

## Discussion

This is, to the best of our knowledge, the first prospective study on the detailed morphology of the walls of intracranial cysts, diagnosed as arachnoid cysts (ACs) from clinical criteria. The study material is also the largest hitherto reported with 24 patients. The most striking overall results are that (i) only half of the samples had a cyst wall arrangement in good agreement with arachnoid tissue; (ii) four cysts made up a morphologically distinct group with a dominant core of cell-poor dense connective tissue lined by a thin squamous epithelium; and (iii) the architecture and cellular composition of the remaining group was variable with an obvious admixture of various elements of an expected intracerebral origin or location, e.g. ciliated epithelia, glial and neuronal components.

None of these findings is unique, a number of case reports exist based on few patients that demonstrate the variability of the arachnoid cyst entity [21-23,25]. Thus, the present study extends and confirms the notion that clinical ACs are a heterogeneous group of pathological conditions. The origin and cause(s) of cyst formation as well as the pathogenic mechanisms behind their expansion into clinically evident cysts have attracted interest over decades [26]. It is noteworthy, that our thorough penetration of patient histories gave no indication as to causative mechanisms behind cyst development. This observation, which is in harmony with previous reports, supports *per se* that these cysts are spontaneous and formed at an early stage, probably as a teratological event [27-29]. It is conceivable that cysts lined and composed of cells characteristic of normal arachnoid originate as errors in the normal splitting of the perineural mesenchymal layers that are destined to form the meninges during early nervous system development. Although there are reports on genetic mechanisms behind AC in combination with more severe encephalic disturbances [30-37] it is not likely that isolated cysts in otherwise healthy patients could be linked to specific genetic errors.

The fibrous cysts in the present report in one respect share morphology with normal dura through the dominance of densely organised cell-poor connective tissue. It might thus be assumed that they reflect erroneous positioning of mesenchymal islands destined for development into dura mater. Experimental evidence based on transgenic techniques indicates that the barrier-forming arachnoid and adjacent dural layers differentiate from a common cell lineage expressing prostaglandin D2 synthase (PGD2S) of neural crest origin at forebrain level and mesodermal origin from midbrain level and posteriorly [38]. In addition, inactivation of the neurofibromatosis type 2 gene during a critical embryonic and early postnatal time window gave rise to meningioma development in PGD2S -positive cell layers with a meningothelial or fibroblastic histological character. These neoplasm types were in continuity with the respective arachnoid and dural layers [38]. It seems justified to speculate that the cited observations on meningioma pathogenesis could have bearing on the developmental mechanisms for cyst formation.

An alternative interpretation of the fibrous cysts is that instead they reflect an equivalent of mature scar or capsule tissue, for example after an early and limited hemorrhage. A recent study presented a “2 hit” process for development of arachnoid cysts with both a congenital histological defect in the development of the arachnoid membrane and a later event of head trauma or hemorrhage [39,40]. This type of mechanism was not evident for the patients in our study, none of whom reported a complication during birth or early childhood and only a few of them reported head trauma.

Cyst-lining epithelial cells equipped with cilia or numerous microvilli indicate a possible origin from ependymal and choroid lineages, respectively. Similarly, the presence of nervous tissue in the group of aberrant extracerebral cysts, anatomically connected with the arachnoid, reasonably represents true malpositioning of neural tube progenitor cells. We lack knowledge of relevant reference literature in the neuro-developmental field that would give clues on possible pathogenetic mechanisms behind such failures in the control of normal development. In a few case reports, authors discuss the possibility that CNS cysts lined by strongly-ciliated epithelia, due to the similarity to respiratory epithelium, are even derived from endodermal progenitors [41,42]. From morphological criteria, the ciliated epithelia encountered in the present material are best compared with the ventricular ependymal lining. Particularly, the simultaneous presence of cilia and numerous long and slender microvillus-like projections are characteristic of ependymal cells [43]. Retrospectively, we realize that the enigma of cell lineage could have been further elucidated by immunohistological analyses, e.g. seeking expression of cytokeratins, gfap, and CD99, to distinguish ependyma from respiratory-like lining. This was not possible in this study due to lack of suitable material.

Regionally the ciliated epithelia also presented a probable secretory morphology that differed from normal ependyma. Whether or not such a differentiation would result in effects on cyst fluid composition or turnover is beyond the scope of the present study. Nevertheless, this finding points at the possibility that cyst wall epithelium can actively modify cyst fluid. This hypothesis is also supported in a recent study by Berle *et al*. [44]. We plan to extend the study and try to find correlates between morphology, fluid chemistry, and clinical properties, including postoperative events, of the various cyst types.

The examined tissue samples invariably had a tight and continuous epithelial lining with seemingly ordered junctions between cells. This would implicate that the luminal epithelium, the primary exchange barrier, limits free exchange of cyst fluid by restricting the possibility for paracellular flow. The degree of resistance should, on the other hand, vary considerably between the attenuated blood capillary endothelium-like junctions of the lowest single layered epithelium, and the elaborate intercellular pathways with multiple specific junctional elements of more complex epithelia. In addition, the variations of stromal thickness and composition including vascularization, and of the arrangement of cells at the dural aspect of cysts, must influence the net fluid exchange properties of cyst walls. Recent studies have focused interest on active mechanisms for cyst fluid accumulation. For example, Berle *et al*. [45] reported that cyst fluid had a composition distinct from cerebrospinal fluid which would require active transport mechanisms. However, considering the drastically different cellular architecture of cyst walls reported here it seems unjustified to expect that cyst fluid is a unitary entity. As an example, assuming that the various epithelia described preserve functional activities characteristic of their normal location, a cyst lined with choroid-like epithelium should conceivably have the potential for fluid formation resembling CSF production [46] whereas the simplest epithelia should lack such activity. Obviously, these considerations urge for further elucidation of possible correlates between cyst fluid contents and cyst wall morphology.

All patients in this study were operated with microsurgical decompression through craniotomy. It is well known that intracranial cysts can re-occlude years after surgery requiring secondary surgery [47,48]. To our knowledge risk factors for reoccurrence have so far not been identified. The cyst morphology could be of importance for long-term surgical outcome. Accordingly, the present work may provide a valuable basis for analysis of cases that might present as future relapses.

## Conclusions

This study on 24 patients established that arachnoid cysts have variable organization of epithelial linings and extracellular components suggesting different barrier properties and fluid turnover characteristics. Further studies are needed to elucidate relations between cyst morphology, cyst pathogenesis, and clinical behavior, including growth rate and relapse tendency. Cyst morphology might be a relevant factor since some cysts become symptomatic [49-51] while others are asymptomatic for years [52-54].

## Competing interests

Carsten Wikkelsø receives honorarium for lecturing and consulting by Codman, Johnson&Johnson, and Likvor AB.

## Authors’ contributions

KR carried out the compiling of patients’ data, participated in collection and preparation of microscopic specimens, performed light and electron microscopy and interpretation thereof, participated in manuscript preparation and took main responsibility in literature search; MT performed all neurosurgical procedures including initial specimen harvesting and drafted the manuscript; CW initiated the study and was the senior medical officer in charge for the clinical presurgical exploration of patients, drafted the manuscript; BRJ supervised and performed light and electron microscopy, assisted KR in preparation of manuscript, performed graphical work of micrographs. All authors have read and approved the final version of the manuscript.
